# Resistance of leukemia cells to cytarabine chemotherapy is mediated by bone marrow stroma, involves cell-surface equilibrative nucleoside transporter-1 removal and correlates with patient outcome

**DOI:** 10.18632/oncotarget.14981

**Published:** 2017-02-01

**Authors:** Patricia Macanas-Pirard, Richard Broekhuizen, Alfonso González, Claudia Oyanadel, Daniel Ernst, Patricia García, Viviana P. Montecinos, Felipe Court, Mauricio Ocqueteau, Pablo Ramirez, Bruno Nervi

**Affiliations:** ^1^ Department of Hematology and Oncology, UC-Center for Investigation in Translational Oncology (CITO), Faculty of Medicine, Pontificia Universidad Católica de Chile, Santiago, Chile; ^2^ Facultad de Medicina, Universidad San Sebastián, Center for Aging and Regeneration (CARE), Faculty of Biological Sciences, Pontificia Universidad Católica de Chile, Santiago, Chile; ^3^ Facultad de Ciencia, Universidad San Sebastián, Fundación Ciencia y Vida, Santiago, Chile; ^4^ Department of Pathology, Advanced Center for Chronic Diseases (ACCDiS), UC-Center for Investigation in Translational Oncology (CITO), Faculty of Medicine, Pontificia Universidad Católica de Chile, Santiago, Chile; ^5^ Department of Physiology, Millennium Nucleus for Regenerative Biology, Faculty of Biology, Pontificia Universidad Católica de Chile, Santiago, Chile; ^6^ Institute of Medicine, Faculty of Medicine, Universidad Austral de Chile, Valdivia, Región de los Ríos, Chile; ^7^ Millennium Institute on Immunology and Immunotherapy, Pontificia Universidad Católica de Chile, Santiago, Chile

**Keywords:** leukemia, cytarabine-resistance, ENT1, bone marrow stroma, biomarker

## Abstract

The interaction between acute myeloid leukemia cells (AML) with the bone marrow stroma cells (BMSCs) determines a protective environment that favors tumor development and resistance to conventional chemotherapy. We showed that BMSCs secrete soluble factors that protect AML cells from Ara-C induced cytotoxicity. This leukemia chemoresistance is associated with a decrease in the equilibrative nucleoside transporter (ENT1) activity by inducing removal of ENT1 from the cell surface. Reduction of cell proliferation was also observed with activation of AKT and mTOR-dependent cell survival pathways, which may also contribute to the tumor chemoprotection. Analysis of primary BMSC cultures has demonstrated that AML patients with stroma capable to confer Ara-C resistance *in vitro* compared to AML patients without this stroma capacity were associated with a worse prognosis. The two year overall survival rate was 0% versus 80% respectively (p=0.0001). This is the first report of a chemoprotection mechanism based on the removal of a drug transporter from the cell surface and most importantly the first time that a stroma phenotype has correlated with prognostic outcome in cancer.

## INTRODUCTION

Acute myeloid leukemia (AML) is a group of neoplastic disorders characterized by the clonal accumulation of immature myeloblasts in the bone marrow (BM) and peripheral blood (PB). The treatment of AML has improved substantially over the past few decades, although the prognosis continues to be relatively poor. Development of resistance to chemotherapy remains a significant problem following conventional chemotherapy treatment when residual disease in the BM relapses into a more resistant leukemia [1]. The most effective agent for the treatment of AML is cytarabine (Ara-C), which is the cornerstone of all standard chemotherapy regiments [2]. Ara-C is a nucleoside analogue which functions by inhibiting DNA synthesis. It is incorporated into cancer cells using specialized membrane transport proteins [3], the human equilibrative nucleoside transporter-1 (ENT1) responsible for 80% of Ara-C uptake in human leukemia cells [4].

The tumor microenvironment, in particular the interaction of leukemia cells with the bone marrow stromal cells (BMSCs), is known to be critical for both cancer development and drug resistance [5, 6]. BMSCs are multipotent mesenchymal stem cells originating from the mesodermal germ layer that gives rise to stromal cells including fibroblasts, adipocytes, chondrocytes and myocytes. All these cell types play a critical role in the regulation of human stem cell maintenance and localization. Recent studies have demonstrated that these ‘BM niches’ stimulate stem cell proliferation, self-renewal, differentiation and trafficking in and out of the BM. BMSCs support both normal and malignant hematopoiesis and produce a wide variety of soluble signals, including cytokines, chemokines and growth factors that activate normal hematopoietic and leukemia precursors. The interaction of leukemia cells with BMSCs contributes to the development of chemotherapy resistance *in vitro* and *in vivo* [5].

We previously reported that mobilization of leukemia cells away from the BM niche into the PB induced by CXCR4 inhibitor AMD3100, increased significantly the overall survival of mice treated with Ara-C [7]. This was likely to be due to the removal of the leukemia cells from the stromal-cell derived chemoprotection. We have also demonstrated that BMSCs provided specific preferential protection to murine leukemia cells from Ara-C induced apoptosis *in vitro*. It is also known that BMSC-induced chemoprotection involves production of soluble factors by the BM stroma which inhibits ENT1 activity [7, 8]. In this study we extended our previous observations to human BMSCs and addressed the potential mechanisms and the prognostic value of the chemoprotection induced by BMSCs from AML patients to the leukemia cells treated with Ara-C.

## RESULTS

### Human bone marrow stromal cell supernatant protects leukemia cell lines from Ara-C induced cytotoxicity

We have previously shown that murine BMSCs supernatant (SN) (from a cell line and primary cells) contains soluble factors that protect murine leukemia cells from Ara-C induced cytotoxicity *in vitro*. We also demonstrated that *in vivo* administration of CXCR4 antagonist, AMD3100, and Ara-C significantly prolonged survival of leukemic mice compared to mice treated with Ara-C alone [7, 8]. These initial findings highlighted the important role of the BM niche in leukemia chemoresistance.

**Figure 1:**
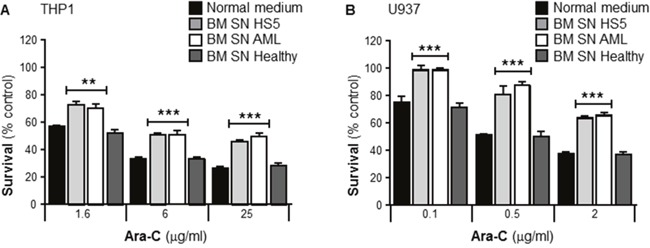
Primary human bone marrow stroma cell supernatant protects leukemia cells from Ara-C induced cytotoxicity Human AML cells lines THP1 **(A)** and U937 **(B)** were cultured in absence or presence of either normal medium (RPMI), human BMSC SN from HS5 (BM SN HS5, a human BMSC cell line), primary human BMSC SN from AML patient (BM SN AML) and primary human BMSC SN from a healthy volunteer (BM SN Healthy) for 2 hours before treatment with Ara-C (1.6, 6 and 25 μg/ml) (A) or Ara-C (0.1, 0.5 and 2 μg/ml) (B) for 24 hours. Leukemia cell viability was assessed by the MTT assay. Each bar represents the mean ± SD of 3 independent experiments. **p < 0.01, ***p < 0.001 (AML cells versus AML cells + human BM SN).

In order to test whether SN from human BMSCs could modify the chemosensitivity of leukemia cells, human leukemia cells lines THP1 and U937 were cultured with or without human BMSC SN from HS5, primary BMSC SN from AML patients or primary BMSC SN from healthy donors. Cells were incubated with Ara-C for 24 hours and cell viability measured using the MTT assay. Figure 1A and 1B demonstrate that both human AML cell lines were significantly chemoprotected by BM SN from HS5 and AML patients from Ara-C induced cytotoxicity, whereas neither BM SN from a healthy volunteer, or normal medium (RPMI) conferred chemoresistance. These data demonstrate that also primary BMSCs from AML patients secrete soluble factors that protect leukemia cells from Ara-C treatment.

### Human bone marrow stromal cells supernatant protects human primary leukemia cells from Ara-C induced cytotoxicity

**Figure 2:**
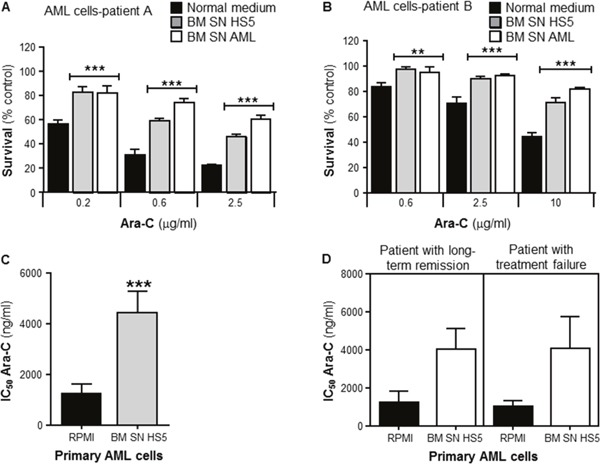
Primary human bone marrow stroma cell supernatant protects human primary leukemia cells from Ara-C induced cytotoxicity Purified human primary leukemia cells from Patient **(A)** and Patient **(B)** were cultured in absence (normal medium) or presence of human BMSC SN from HS5 (BM SN HS5) or primary human BMSC SN from AML patient (BM SN AML) for 2 hours before treatment with Ara-C (0.2, 0,6 and 2.5 μg/ml) (A) or Ara-C (0.6, 25 and 10 μg/ml) (B) for 72 hours. Leukemia cell viability was assessed by the MTT assay. Each bar represents the mean ± SD of 3 independent experiments. **p < 0.01, ***p < 0.001 (leukemia cells versus leukemia cells + human BM SN). **(C)** Overall analysis of IC_50_s for Ara-C from primary leukemia cells obtained from 20 AML patient samples incubated in normal culture medium (RPMI) or human BMSC SN from HS5 (BM SN HS5) treated with Ara-C for 72 hours. ***p < 0.001 (RPMI IC_50_s versus SN IC_50_s). **(D)** Overall analysis of primary leukemia IC_50_s from 18 AML patient samples with long-term remission versus patients with treatment failure. Primary leukemia cells were incubated in normal culture medium (RPMI) or BM SN HS5 treated with Ara-C for 72 hours.

To investigate whether human BMSCs could also confer Ara-C resistance to human primary leukemia, cells from newly diagnosed AML patients were collected and purified. These primary leukemia cells were incubated with or without human BMSC SN from HS5, or primary BMSC SN from AML patients. Patient samples were incubated with Ara-C for 72 hours before cell viability was measured by the MTT assay. Figure 2A and 2B show data from 2 newly diagnosed representative AML patients. Primary human leukemia cells from both patients were significantly chemoprotected by human BMSC SN from HS5 and primary BMSC SN from AML patients from the cytotoxic effects of Ara-C. Combined data from n=20 AML patients (each patient leukemia cells were tested for Ara-C sensitivity with HS5 SN) showed that Ara-C IC_50_ values were significantly higher in primary leukemia cells cultured with HS5 SN compared with leukemia cells cultured in normal medium (RPMI), demonstrating HS5 SN mediated chemoprotection (Figure 2C). Furthermore, as observed in Figure 2D, Ara-C patient leukemia sensitivity for both groups (RPMI and HS5 SN) showed no significant difference in the clinical outcome for patients with long-term remission versus patients with treatment failure. There was no evidence that the variation of Ara-C sensitivity of primary leukemia cells was a prognostic survival factor for patients with AML. Overall, we found that neither the *in vitro* primary leukemia Ara-C sensitivity (IC_50_), nor the magnitude of the leukemia resistance, correlated with any clinical outcome investigated (remission induction, relapse, or overall survival (data not shown)).

### Bone marrow stromal cell supernatant from AML patients collected both at diagnosis and in remission, protect leukemia cells from Ara-C induced cytotoxicity

**Figure 3:**
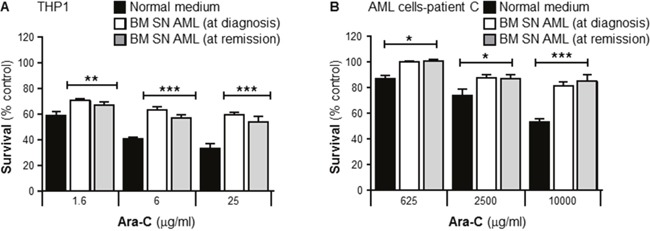
Primary human bone marrow stroma cell supernatant collected from a leukemia patient in remission protects primary leukemia cells from Ara-C induced cytotoxicity THP1 cells **(A)** and purified human primary leukemia cells from a patient with AML (Patient C) **(B)** were cultured with or without primary human BMSC SN collected from the patient before and after chemotherapy treatment and confirmed remission, for 2 hours before treatment with Ara-C (1.6, 6 and 25 μg/ml) for 24 hours (THP-1 cells) or Ara-C (625, 2500 and 10000 μg/ml) for 72 hours (primary leukemia cells). Leukemia cell viability was assessed by the MTT assay. Each bar represents the mean ± SD of 3 independent experiments. *p < 0.05; **p < 0.01 ***p < 0.001 (leukemia cells in normal medium (RPMI) versus leukemia cells in patient's BM stroma).

It is not known whether the capacity of BMSCs to confer Ara-C resistance *in vitro* depends on the direct interaction leukemia-stroma, or if there is a stable stroma transformation into a “malignant or activated” stroma. To evaluate if the stroma capacity to confer resistance could be affected after an effective induction treatment with Ara-C based chemotherapy (complete remission; less than 5% leukemia blasts at 30 days post chemotherapy treatment, following complete hematopoiesis recovery). THP1 cells and human primary leukemia cells were incubated with or without human BMSCs SN from AML patients at diagnosis or remission. Both cultures from the same patients were incubated with Ara-C for 24 or 72 hours respectively and cell viability measured using the MTT assay. Figure 3A and 3B shows that THP1 cells and primary human leukemia cells were significantly protected by both AML BMSC SNs (at diagnosis and remission) from the cytotoxic effects of Ara-C. These results demonstrated that chemotherapy treatment has no effect on the BMSCs capacity to protect leukemia cells.

### Investigating the mechanism of leukemia cell protection from Ara-C induced by BMSCs

#### Bone marrow stromal cell supernatant potency

**Figure 4:**
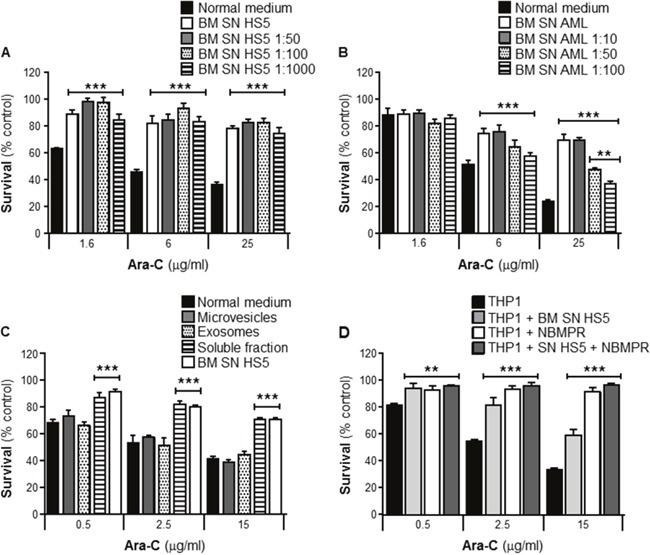
BMSCs secrete potent soluble factors that protect leukemia cells from Ara-C induced cytotoxicity THP1 cells were cultured with or without human BMSC SN from HS5 (BM SN HS5) undiluted or diluted (HS5 1:50, 1:100 and 1:1000) **(A)**, primary human BMSC SN from an AML patient undiluted or diluted (BM SN AML 1:10, 1:50 and 1:100) **(B)** for 2 hours before treatment with Ara-C (1.6, 6 and 25 μg/ml) for 24 hours. BM SN HS5 was processed by ultracentrifugation to purify microvesicles, exosomes and a soluble protein fraction (P10, P100 and S100) for 2 hours before treatment with Ara-C (0.5, 2.5 and 15 μg/ml) for 24 hours **(C)** Leukemia cell viability was assessed by the MTT assay. Each bar represents the mean ± SD of 3 independent experiments. **p < 0.01, ***p < 0.001 (leukemia cells in normal medium versus leukemia cells BMSC SNs). **(D)** THP1 cells were cultured alone or with human BMSC SN from HS5 (BM SN HS5) with pre-treatment with NBMPR (1μM) for 2 hours before normal treatment with Ara-C (0.5, 2.5 and 15 μg/ml) for 24 hours. Cell viability was measured by the MTT assay. Data are the mean ±SD of at least three independent experiments. ***p < 0.001 (THP1 versus THP1 + NBMPR).

Several reports have shown that BMSCs secrete a wide variety of soluble factors that protect leukemia cells from the cytotoxic effects of Ara-C [7–9]. In order to investigate the potency of this chemoprotective effect, survival assays from serial dilutions were performed. THP1 cells were cultured with or without human BMSC SN from HS5 neat or diluted (BM SN HS5 1:50, 1:100 and 1:1000), with or without primary human BMSC SN from an AML patient neat or diluted (BM SN AML 1:10, 1:50 and 1:100). Cells were incubated with Ara-C for 24 hours and cell viability was measured by the MTT assay. Figure 4A showed that THP1 cells were protected as effectively from Ara-C toxicity by HS5 SN diluted 1:1000, as they were by neat HS5 SN. Figure 4B, showed THP1 chemoprotection from Ara-C by primary BMSCs SN was effective when used neat or diluted 1:10, 1:50 and 1:100.

### Bone marrow stromal cell supernatant composition

In addition to soluble proteins, cells also release membrane vesicles in the extracellular environment for intercellular communication [10]. To evaluate whether secreted extracellular vesicles contribute to drug resistance of leukemia cells, three SN fractions were prepared by differential centrifugation of freshly prepared HS5 SN; microvesicles (P10), exosomes (P100) and mainly soluble proteins (S100). THP1 cells were incubated for 2 hours with these fractions obtained from the HS5 stroma cell line before treatment with Ara-C for 24 hours. In contrast to the chemoprotection provided by both HS5 SN and the fraction containing soluble proteins (such as cytokines, chemokines and growth factors), neither microvesicles nor exosomes fractions had a significant effect (Figure 4C). These data indicate that soluble factors rather than vesicles secreted by BMSCs account for leukemia resistance against Ara-C toxicity.

### Bone marrow stromal cell supernatant chemoprotection can be replicated by inhibiting the ENT1 pathway

Transport of Ara-C across the cell membrane is primarily dependent on ENT1 [11]. To investigate whether ENT1 is a key player for Ara-C chemoprotection observed when leukemia cells are incubated with HS5 SN, THP1 cells were incubated with human BMSC SN from HS5 in the presence or absence of a selective ENT1 small molecule inhibitor, Nitrobenzylthioinosine (NBMPR). NBMPR (1μM) was added 2 hours before treatment with Ara-C for 24 hours. Cell viability was measured using the MTT assay. Figure 4D shows that inhibition of ENT1 transporter by NBMPR conferred a significant chemoprotection to Ara-C when administered in the absence of HS5 SN. These data suggest that soluble factor(s) derived from BMSCs may inhibit ENT1 activity resulting in resistance of leukemia cells to the cytotoxic effects of Ara-C associated with a reduced intracellular incorporation.

### Human bone marrow stroma cell conditioned medium inhibits leukemia cell cycle/proliferation

**Figure 5:**
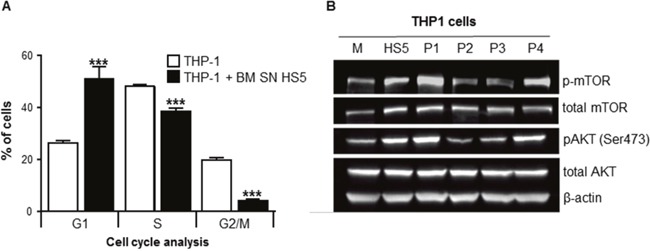
BMSCs SN arrested leukemia cell cycle/proliferation and activated AKT and mTOR intracellular signaling THP1 cells were cultured with or without human BMSC SN from HS5 (BM SN HS5) for 24 hours and incubated overnight with BrdU. Cells were harvested, fixed and stained with 7-AAD and analyzed using flow cytometry. **(A)** THP1 cells were shown to be at the different stages of the cell cycle (G_1_, S and G_2_/M). **(B)** THP1 cells were incubated in normal growth medium (M) or human BMSC SN from HS5 (HS5) or primary BMSCs SNs from AML patients (P1-P4) for 24 hours. Cell lysates were processed for western blot analysis to detect human phosphorylated mTOR, total-mTOR, phosphorylated AKT, total AKT and β-actin (loading control). Each bar represents the mean ± SD of 3 independent experiments. ***p < 0.001 (THP-1 versus THP1 SN).

To explore mechanisms of BMSC-induced chemoprotection to Ara-C, the effect on cell proliferation was investigated. THP1 cells were cultured with or without human BMSC SN from HS5 (BM SN HS5), stained with Bromodeoxyuridine (BrdU) and 7-aminoactinomycin D (7-AAD) in order to analyze the cell cycle using flow cytometry. HS5 SN caused a significant accumulation of THP1 cells in G_0_/G_1_ phase (cell cycle arrest), decreased the fraction of proliferating (S-phase) and G_2_/M cell division phase of THP1 cells (Figure 5A). The increased proportion of cells in G_0_/G_1_ indicates a cell cycle arrest. These results highlight the role of the BMSCs in the induction of quiescence in leukemia cells within the BM microenvironment, which might contribute to chemoprotection from cell-cycle dependent cytotoxic agents such as Ara-C.

### Human bone marrow stromal cell supernatant activates AKT and mTOR

The effects of HS5 SN on signaling pathways involved in cell survival, such as AKT and mTOR were investigated [12]. THP1 cells treated with human BMSC SN from HS5 had increased levels of total mTOR and AKT proteins compared with cells incubated in normal growth media (Figure 5B). The activation (phosphorylation) of mTOR and AKT was also explored in THP1 cells incubated with normal growth medium (M) BM SN from HS5 (HS5) or primary BMSC SN from AML patients (Patients P1-P4). BMSC SNs from patients P1 and P4 have a “protective” phenotype (stroma cells from AML patients that confer chemoresistance to Ara-C) whereas P2 and P3 SNs have a “non-protective” phenotype. THP1 cells incubated with HS5, P1 and P4 SNs showed activation of AKT and mTOR compared to normal growth medium (M), P2 and P3 SNs (non-protective). These results indicate that primary BMSC SNs from AML patients with a “protective” phenotype activate AKT and mTOR intracellular pathways involved in cell survival.

### Bone marrow stromal cell supernatant-induce Ara-C chemoprotection by removing ENT1 from the cell surface

**Figure 6:**
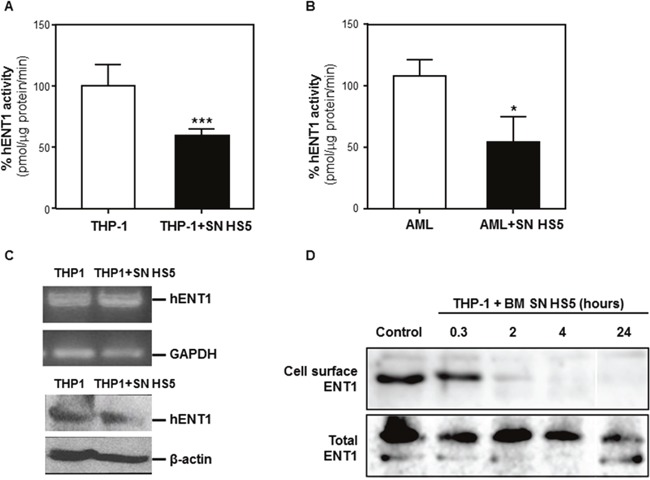
BMSCs secrete soluble factor(s) that decrease ENT1 nucleotide transport protein activity by removing ENT1 from the cell surface THP-1 cells **(A)** or AML patient samples **(B)** were cultured alone or with human BMSC SN from HS5 (SN HS5) for 24 hours. Cells were harvested and hENT1 activity was measured by the incorporation of radioactive ^3^H-adenosine. Each bar represents the mean ± SD of 3 independent experiments. ***p < 0.001 (THP1 versus THP1 in HS5 SN). *p < 0.05 (patient AML versus patient AML cultured HS5). **(C)** RNA extraction from THP1 cells cultured alone or in presence of human BMSC SN from HS5 (SN HS5) for 24 hours was used for RT-PCR to detect expression of ENT1. **(D)** The ENT1 protein from THP1 cells disappears from the cell surface by BMSC SN HS5 treatment. TPH1 cells were incubated with human BMSC SN from HS5 (BM SN HS5) for the indicated time periods at 37°C and were subjected to cell surface biotinylation at 4°C. The biotinylated proteins were then precipitated using Neutroavidin-Sepharose, resolved in SDS-PAGE and analyzed by immunoblot with anti-ENT1 antibodies. Total ENT1 corresponds to the immunoblot of 20% of the total cell extract. BM SN HS5 treatment induced removal of ENT1 from the cell surface in the first 2 hours and extending to the 24 hours of the experiment, without major changes in the ENT1 molecular mass.

Transport of Ara-C across the cell membrane is primarily dependent on ENT1 and the ability of BMSC SN to decrease ENT1 activity in murine leukemia cells has been previously demonstrated [8]. In order to quantify ENT1 transporter activity in these experiments, analysis of ^3^H-adenosine incorporation into THP1 cells, cultured with or without human BMSC SN from HS5 for 24 hours, was assessed. HS5 SN decreased ENT1 activity 40% compared to leukemia cells incubated in medium (Figure 6A). A significant reduction (50%) in ENT1 activity was also observed in primary AML cells incubated with HS5 SN (Figure 6B). Importantly, these effects were not due to changes in the expression of ENT1 at the mRNA or protein level (Figure 6C), suggesting a post-translational mechanism.

To investigate whether HS5 SN affects the expression of ENT1 at the cell surface we performed an ENT1 extracellular domain biotinylation assay [13]. This assay detected ENT1 at the cell surface and its levels decreased after 2 hours of incubation with HS5 SN, became undetectable after 4 hours and remained undetectable at 24 hours. There were no major changes in the molecular mass of the transporter (Figure 6D). These results indicate that HS5 SN induced a rapid and prolonged removal of ENT1 from the cell surface.

Taken together, these results indicate that soluble factor(s) derived from BMSCs induce a removal of ENT1 from the cell surface, resulting in lower nucleoside transporter activity, which may be responsible for the subsequent resistance of leukemia cells to the cytotoxic effects of Ara-C.

### The capacity of patients BMSCs to confer resistance to Ara-C *in vitro* is a prognostic factor for overall survival

As BMSCs induced chemoresistance to Ara-C in human THP-1 leukemia cells *in vitro*, we then investigated whether the AML patient BMSC capacity to confer resistance *in-vitro* correlated with clinical outcomes. We obtained BM aspirate samples from 18 patients presenting with AML, expanded their stroma, studied their capacity to confer Ara-C chemoresistance *in vitro*, and analyzed their correlation with overall survival. Table 1 details the main clinical and diagnostic laboratory data of all 18 patients.

**Table 1:** AML patient characteristics included in this study (n=18)

#	Age	Risk factor	Risk	IC_50_ control	IC_50_ SN	resistance factor	Induction chemotherapy	Induction remision	Consolidation chemotherapy	Allogeneic SCT	Relapse	Survival (days)	Status
1	38	NPM1 mut	low	3,8 ± 0,8	2,4 ± 1,5	**0.6**	7+3	CR	HiDAC x 2	no	no	548	alive
2	29	t(16;16)	low	2,8 ± 0,8	1,0 ± 0,2	**0.4**	7+3	CR	HiDAC x 2	no	no	484	alive
3	57	t(8;21)	low	3,8 ± 0,8	3,2 ± 1,3	**0.8**	7+3	CR	HiDAC x 2	no	no	547	alive
4	56		intermediate	4,7 ± 0,4	1,8 ± 0,2	**0.4**	7+3	CR	HiDAC x 2	no	yes	564	death
5	55		intermediate	4,3 ± 0,7	2,8 ± 1,1	**0.7**	7+3	CR	HAM	no	no	1137	alive
6	42		intermediate	4,3 ± 0,8	16,6 ± 0,8	**3.9**	7+3	CR	HAM x 2	no	yes	336	death
7	62	> 60 years	high	4,7 ± 0,4	1,9 ± 0,1	**0.4**	7+3	CR	HiDAC x 2	no	no	1124	alive
8	45	complex cariotipe	high	2,6 ± 0,6	1,2 ± 0,1	**0.5**	7+3	CR	Azacitidina x 6	no	yes	227	death
9	37	> 30,000 blast	high	4,1 ± 1,2	3,0 ± 1,5	**0.7**	7+3	CR	FLAG-IDA, HiDAC x 2	no	no	781	alive
10	26	complex cariotipe	high	3,1 ± 0,1	2,6 ± 0,1	**0.8**	7+3	<5% blast	FLAG-IDA, MEC	yes	no	735	alive
11	69	> 60 years	high	3,1 ± 0,1	2,8 ± 0,3	**0.9**	7+3	CR	Azacitidina x 4	no	yes	960	alive
12	61	> 60 years	high	3,4 ± 0,1	3,7 ± 0,1	**1.1**	No	no	no	no	yes	292	death
13	74	> 60 years	high	4,3 ± 0,7	16 ± 0,4	**3.7**	No	no	no	no	yes	287	death
14	48	> 30,000 blast	high	4,3 ± 0,7	16 ± 0,4	**3.7**	7+3	CR	HiDAC x 3	no	yes	244	death
15	62	> 60 years	high	4,5 ± 0,6	45 ± 1,2	**10.2**	7+3	CR	Azacitidina x 6	no	yes	357	death
16	33	AML 2nd relapse	high	4,0 ± 0,4	94 ± 0,4	**23.5**	FLAG-IDA	CR	HiDAC x 1	yes	yes	523	death
17	37	complex cariotipe	high	3,4 ± 0,1	102 ± 0,4	**30.0**	7+3	<5% blast	HiDAC x 1	no	yes	52	death
18	85	> 60 years	high	2,1 ± 0,1	67,3 ± 0,4	**32.0**	No	no	no	no	yes	45	death

Abbreviations: IC_50_ control, IC_50_ using normal growth medium; IC_50_ SN, IC_50_ using supernatant from BMSCs; NPMI^1^, Nucleophosmin; RF^2^, Resistance factor; Chemo^3^, chemotherapy; 7+3^4^, Ara-C (7 days) and Daunorrubicin (3 days); CR^5^, complete remission; HiDAC^6^, High-dose Ara-C; t(16;16)^7^, translocation (16;16); Int^8^, intermediate; HAM^9^, High-dose Ara-C and Mitoxantrone. FLAG-IDA^10^, Fludarabine, Ara-C, Idarrubicin and G-CSF; MEC^11^, Mitoxantrone, Etoposide and cytarabine (Ara-C).

**Figure 7:**
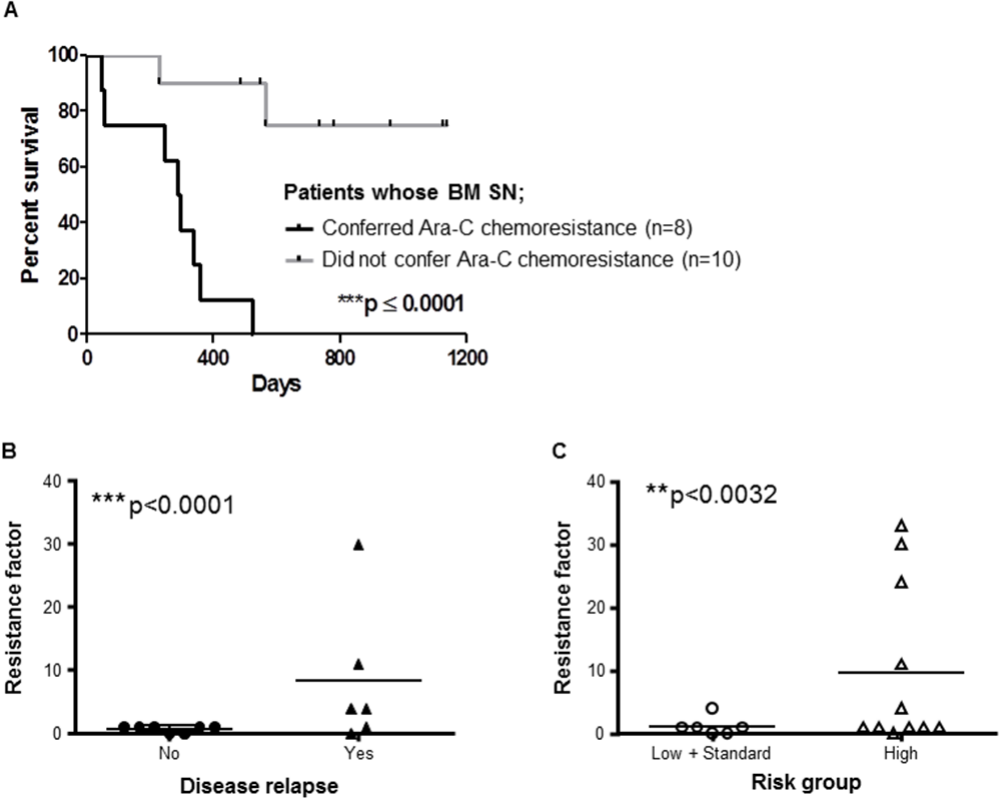
BMSCs from AML patients play key role in patients’ outcome **(A)** Kaplan Meier analysis of overall survival of n=18 patients with AML whose BMSCs were isolated, cultured and used for chemosensitivity tests with THP1. THP1 cells were cultured with or without primary human BMSC SN from AML patients for 2 hours before treatment with Ara-C (0.2, 0.6 and 2.5 μg/ml) for 48 hours. THP1 cell viability was assessed by the MTT assay to obtain IC_50_. Data are the mean ±SD of at least three independent experiments. ***p < 0.0001 (patients with protective BMSCs versus patients with non-protective BMSCs). **(B)** Stroma protection factor versus relapse status of AML patients. The stroma protection factor was calculated as the IC_50_ of THP1 cells incubated with primary BMSC SN from AML patients/ IC_50_ of THP-1 cells incubated with normal growth medium. Relapse was defined as bone marrow blasts ≥5%; or reappearance of blasts in the blood; or development of extramedullary disease in 13 AML patients according to Döhner et al. Blood; 2010 [43]. ***p < 0.001 (relapse: no versus yes). **(C)** Stroma protection factor according to AML risk. The stromal protection factor was calculated as the IC_50_ of THP1 cells incubated with primary BMSC SN from AML patients/ IC_50_ of THP1 cells incubated with normal growth medium. N=17 patients with AML were stratified according to their risk group (low, standard or high) [44] **p < 0.0032 (low and standard versus high risk AML patients).

The capacity of BMSC SNs from 18 AML patients (treated with conventional Ara-C based chemotherapy) to confer resistance to Ara-C *in vitro* was investigated using the THP1 cell chemosensitivity assay. In n=8 patients (44.4%), the BM SN conferred resistance defined as an increase in the Ara-C IC_50_ >100% in the THP1 survival assay (IC50 means the Ara-C concentration able to kill 50% of tumor cells). We defined the “resistance factor” as the Ara-C IC_50_ generated using the conditioned media divided by the equivalent control IC_50_. For example, a resistance factor of 2 means that the stroma SN confers Ara-C resistance requiring a 100% dose increase for the same activity. Furthermore, Kaplan-Meier survival analysis demonstrated that BMSC SNs from these patients had a significant poor survival outcome compared to patients with BMSC SNs that did not confer chemoresistance (Figure 7A). The 2 years overall survival rate was 0% versus 80% respectively, with a median follow up of 513 days. The median survival was 290 days in the survival group versus not reached in the second group (HR 0.06 CI 0.014-0.257, ^**^*p <0.0001). In the examination of the stroma cells from patients with AML, the stroma phenotype that conferred resistance to Ara-C in the *in vitro* assay correlated with overall survival, as previously observed (Figure 7A), and also with disease relapse, the major risk factor for AML survival (p< 0.0001; Figure 7B). These results indicate that the BMSC capacity to induce resistance of leukemia cells to Ara-C is a major risk factor for relapse and overall survival. This information was compared with the prognostic risk factors conventionally studied for the stratification of patients into low-, intermediate- and high-risk AML (including age, leukocytosis >30,000 cells/mm^3^, complex karyotype and low risk translocations t(15;17), t(8;21), inv16; and high risk FLT3 ITD mutation). Interestingly, those patients with BM stroma capable to confer resistance to Ara-C *in vitro* are also of high-risk according to the conventional risk factors (p<0.0032; Figure 7C) (Table 1).

In summary, these results presented here describe the development of an *in vitro* assay that determines the capacity of AML patient stroma to confer Ara-C resistance, as a strong risk factor for the clinical outcomes relapse and overall survival.

## DISCUSSION

Compelling evidence supports the hypothesis that the interaction between leukemia cells with the BM stroma results in a protective environment favoring tumor development and resistance to cytotoxic treatments [14–17]. We investigated the mechanisms involved in BMSCs mediated AML chemoresistance to identify potential predictive markers for clinical response to Ara-C treatment.

BMSCs SN from both the HS5 cell line and primary BMSCs (about 50% of cases) was shown to protect leukemia cells from Ara-C induced cytotoxicity *in vitro*. These data support our previous findings using an *in vivo*/*in vitro* mouse AML model [7, 8].

The HS5 cell line is a human marrow stromal cell line immortalized by transduction with the papilloma virus E6/E7 that supports proliferation of hematopoietic progenitor cells when co-cultured in serum-deprived media with no exogenous factors. They produce significant levels of granulocyte colony-stimulating factor (G-CSF), granulocyte-macrophage-CSF (GM-CSF), macrophage-CSF (M-CSF), Kit ligand (KL), macrophage-inhibitory protein-1a, interleukin-6 (IL-6), IL-8, and IL-11[18]. Even a 1000-fold dilution of HS5 and a 100-fold dilution of primary AML BMSC SNs preserved the capacity to confer Ara-C chemoresistance to leukemia cells. This suggests that BMSCs secrete soluble factors which are highly potent in protecting leukemia cells from Ara-C treatment.

An inter-cellular communication method which has recently been described, involves secretion of membrane fragments classified as microvesicles or exosomes according with the isolation procedure [19, 20]. Previous work by Wang et al. showed that BMSCs communicate with multiple myeloma cells through exosomes which carry selective cytokines, favoring tumor proliferation, migration, drug resistance and survival [21]. However, in our experiments, SN fractions containing microvesicles or exosomes caused no protection of THP1 cells from the cytotoxic effects of Ara-C, in contrast to the fraction containing BMSCs-secreted soluble proteins. These results provide evidence that the protection conferred by the stroma involves protein factors which are not transferred by microvesicles or exosomes.

The BM stroma cells play a crucial role in drug resistance, control of cell growth and leukemia cell survival [22]. We demonstrated that HS5 SN caused a significant accumulation of leukemia cells in G_0_/G_1_ phase (cell cycle arrest), decreased the fraction of proliferating (S-phase) and G_2_/M cell division phase of THP1 cells. This cell cycle arrest indicates that BMSCs secreted soluble factors can crucially contribute to enhance the accumulation of quiescent leukemia cells within the BM microenvironment. Conversely, cell cycle quiescence is also a known mechanism of stroma-induced resistance to cell cycle-specific chemotherapy agents such as Ara-C [9].

We and others have demonstrated that BMSCs can trigger activation of multiple pro-survival signaling pathways both through secreted soluble factors and by direct cell-to-cell communication [7, 8, 23–27]. Zeng et al. demonstrated that the stroma stimulated the PI3K/mTOR/AKT pro-survival signaling pathway by up-regulating phosphorylation of AKT [28]. We showed that HS5 SN activated phosphorylation of AKT and mTOR indicating an increased activity of these pro-survival kinases in THP-1 cells. The AKT signaling pathway is thought to play an important role in the resistance of BM-resident AML cells and determines the level of residual leukemia cells in AML patients. The molecular mechanisms of AML chemoresistance to Ara-C have been widely investigated and mainly involve the constitutive activation of tyrosine kinase receptors and intracellular signal transduction molecules such as PI3K, ERK, AKT, mTOR [9, 28, 29]. Konopleva et al 2013, proposed that TGF-β secreted by the BM stroma cells plays an important role in AML chemoresistance to Ara-C [9]. Piya et al. has also shown that BM microenvironment autophagy plays an important role in AML chemoresistance [30]. Furthermore, recent reports have focused on the overexpression of the oncogene ERG and also phosphoinositide 4-phosphatase as potential markers for treatment failure in patients with AML [31, 32].

Ara-C continues to be the most effective agent for the treatment of patients with AML. Once inside the cell, Ara-C is phosphorylated to Ara-CTP, which is then incorporated into the DNA causing its cytotoxic effect by inhibition of DNA synthesis. Ara-C resistance is thought to arise from a deficiency in Ara-C phosphorylation by deoxyxytidine kinase (dCK) associated with an increased inactivation of Ara-C by deamination from cytidine deaminase (CDA). However, even though these enzymatic activities may contribute to the resistance of cells to Ara-C still have not shown to be valuable predictors of clinical response to Ara-C treatment [33–36].

The nucleoside transporter ENT1 is responsible for up to 80% of Ara-C influx into human leukemic cells [4, 11]. A deficiency in ENT1 confers high-level of resistance to Ara-C in leukemia cells [37, 38], and a lower expression of ENT1 mRNA has been related with a shorter disease-free survival in patients with AML [39]. One of the most frequent class I mutations in patients with AML is fms-related tyrosine kinase 3-internal tandem duplication (FLT3-ITD), which occurs in about 25% of AML patients. Interestingly, a recent report suggested that the FLT3-ITD specifically induces Ara-C resistance in AML by repressing ENT1 mRNA expression [40]. We showed that inhibition of ENT1 by NBMPR, a selective ENT1 small molecule inhibitor, conferred a significant chemoprotection to leukemia cells from Ara-C. Similarly, our studies showed a significant reduction of ENT1 activity in AML cell line THP1 and primary AML cells incubated with HS5 SN. In-line with previously reported findings in a murine model system [41], no changes in ENT1 mRNA or protein expression was observed in THP1 cells treated with HS5 SN. This suggests that a post-translational process controls ENT1 activity at the cell surface, either by directly modifying the transporter efficiency or inducing its endocytic removal from the plasma membrane. Although the first possibility cannot be ruled-out, this study shows evidence, using a cell surface biotinylation assay, that HS5 SN induces a rapid (within 2 hours) and prolonged (24 hours) removal of ENT1 from the cell surface.

To explore the translational relevance of these observations, the prognostic value of Ara-C chemoresistance conferred by the BM stroma was evaluated in patient samples with AML. Overall analysis demonstrated that the intensity and variation of Ara-C sensitivity (IC_50_) of primary leukemia cells *in vitro* with HS5 SN was not a prognostic survival factor for AML patients (cohort of 20 patients). Furthermore, leukemia sensitivity to Ara-C did not correlate with induction remission 30 days post chemotherapy treatment. On the other hand, primary BMSC SN (cohort of 18 AML patients) *in vitro* chemoprotective effect over THP-1 cells correlated with patient overall survival. Furthermore, only 40% of all AML BMSC SNs tested showed positive chemoprotective effect to leukemia THP-1 cells. It is therefore possible that the stroma-leukemia biology may be even more relevant than the leukemia genetics.

We propose that the stroma probably develops the capacity to confer leukemia resistance to Ara-C *in vivo* induced by tumor cells such as AML and this itself could add additional prognostic information to the standard risk-factors currently in use to stratify the risk of AML patients. This may provide additional information for intermediate-risk patients that do not harbor any known predictive markers or for patients older than 60 years old (have a poor prognosis as a group) who are not normally offered treatment with curative intent. The evaluation of the stroma capacity to confer resistance could enable recognition of a subgroup of patients who may benefit from alternative treatment strategies. This mechanism of resistance conferred by the stroma may be a common mechanism observed in different types of tumors. Remarkably, this BM capacity to confer resistance to Ara-C was preferentially observed in those patients that harbored high-risk prognostic factors associated with poor survival. We propose the hypothesis that the leukemia from high-risk patients is capable of educating the tumor stroma to acquire secondarily the capacity to confer resistance.

In summary, this study demonstrated that human BM stroma secretes soluble factor(s) which assists AML cells in developing resistance to Ara-C. This mechanism seems to occur by ENT1 transporter removal from the cell surface. Interestingly, some AML patients harbor a BM stroma that has acquired the capacity to confer resistance and this stroma phenotype correlates with patient overall survival. This observation requires further study to confirm whether the BM stroma is a prognostic or predictive factor to Ara-C clinical response that may contribute to stratify patient's risk optimizing the treatment approaches.

New strategies to improve current therapies in AML are urgently needed to induce remission and to eliminate residual disease, especially in patients with AML relapse that are incurable with conventional chemotherapy. It would be important to search for specific inhibitors of the BM stroma-resistance pathway, to render existing treatments more effective.

## MATERIALS AND METHODS

### Reagents

Citarabine (Ara-C) was purchased from Pfizer (Bentley, WA, Australia). Nitrobenzylmercaptopurine (NBMPR) was obtained from Sigma Chemical Co. (St. Louis, MO).

### Samples from patients with AML

Primary AML samples from the BM were obtained from patients with newly diagnosed AML who had provided informed consent in accordance with institutional guidelines set forth by the Ethics Committee of the Faculty of Medicine, Pontificia Universidad Católica de Chile (CEI-MEDUC 10-101).

### AML cell culture and purification

The human BMSC line HS5 (HS5 BMSCs) was kindly provided by Dr. John DiPersio (Washington University School of Medicine, St. Louis, USA). The THP1 and U937 AML cell lines were purchased from ATCC (Manassas, VA). All cell lines were cultured in RPMI 1640 (Invitrogen, Carlsbad, CA, USA), supplemented with 10% (v/v) FBS, 100 IU/ml penicillin and 100 μg/ml streptomycin, non-essential amino acids and 2 mm L-glutamine (complete medium) in a humidified incubator at 37°C with 5% carbon dioxide. To prepare supernatant (SN) from HS5, cells were grown for 24 hours in supplemented RPMI to near confluence, centrifuged at 1,000 g for 5 minutes. Cell-free culture SNs was obtained by passage through a 0.45μm sterile filter. For serial dilutions of the BMSC SN, the HS5 or primary BMSC SN was diluted accordingly using fresh RPMI culture medium.

AML BM aspirates collected during standard diagnostic procedures were obtained from the posterior iliac crest and collected in a heparinized syringe. Mononuclear cells were purified by Ficoll-Histopaque density-gradient centrifugation as described by the manufacturers (GE Healthcare Bio-sciences AB, Uppsala, Sweden). Patient samples were re-suspended in complete RPMI 1640 medium and were analyzed by flow cytometry and only patients with ≥50% AML blasts were used for chemosensitivity studies. Mononuclear cells were also re-suspended in DMEM supplemented with 10% FBS, L-glutamine and penicillin-streptomycin mixture followed by plating at an initial seeding density of 1 × 10^6^ cells/cm^2^. After 3 days, the non-adherent cells were removed by washing with phosphate-buffered saline (PBS), and monolayers of adherent cells were cultured until they reached confluence. Cells were then trypsinized, sub-cultured and allowed to grow to near confluence to use for the experiments. Cell-free culture SNs was obtained by passage through a 0.45μm sterile filter. The primary BM SC cultures were negative for markers of the hematopoietic lineage CD14, CD34, and CD45 as analyzed by flow cytometry.

### Treatment of AML cells

Human AML cells cultured alone, cultured with HS5 BMSC SN or primary human BMSC SN were exposed to Ara-C for 48 (human cell lines) or 72 hours (primary AML samples) before cell viability analysis.

### Detection of cell viability

THP1 and U937 AML cells and primary AML cells were cultured in 96-well plates with or without BM SN for 2 hours before treatment with Ara-C for 24 hours. Cell viability was assessed by the MTT (3-(4,5-dimethylthiazol-2-yl)-2,5-diphenyl tetrazolium bromide) assay (Sigma, St. Louis, MO). Two hours before ending the treatment, 10 μl of MTT (5mg/ml saline) was added to each well, the samples were incubated for 2 hours at 37°C. Cells were lysed and MTT crystals solubilized by the addition of 100 μl of 0.02 N HCl in isopropanol. The absorbance of each well was determined at 590nm using a BioTek microplate reader (BioTek instruments, Winooski, VT). Cell viability (%) was calculated relative to the control.

### Cell proliferation assay

THP1 cells grown in 24-well plates with or without HS5 BM SN were incubated with bromodeoxyuridine at various time-points as described by the manufacturers (FITC BrdU flow kit, BD Pharmingen, San Diego, CA). THP1 cells were harvested, fixed and labelled with anti-BrdU fluorescein isothiocyanate antibody and 7-AAD to analyze proliferation by flow cytometry.

### Microvesicle purification

Vesicle purification was performed as previously described [10, 42]. HS5 cells were cultivated in RPMI medium supplemented with 10% exosome-free FBS (obtained by serum ultracentrifugation at 100,000g for 12 h) for 48 hours. The HS5 SN was then subjected to serial centrifugations at 300g for 10 minutes (pellet discarded), 3000g for 10 minutes (pellet discarded); followed by 10,000g for 30 minutes at 4°C and by ultracentrifugation at 100,000g for 70 minutes at 4°C. Different pellets named P10 (large vesicles 1-5 μm diameter) (after the 10,000g centrifugation) and P100 (exosomes) (after 100,000g ultracentrifugation) were washed in cold 0.1M phosphate buffer saline, pH 7.4 (PBS) and centrifuged again at respectively 10,000g or 100,000g. Both pellets were re-suspended in RPMI medium. The 100,000g ultracentrifugation SN was named S100 and predominantly contained soluble proteins.

### Protein extraction and western blot

Cell pellets were washed twice in ice cold PBS and lysed with lysis buffer and protease inhibitors (20 mM Tris (pH 7.5), 1% triton, 10% glycerol, 137 mM NaCl and 2 mM EDTA, 250 μM PMSF, 5μg/ml leupeptin) for 20 min, followed by centrifugation at 10,000 rpm for 10 min at 4°C. Protein concentration of the SN was measured using Bio-Rad protein assay dye reagent (Bio-Rad, Hercules, CA, USA). Fifty micrograms of proteins were equally loaded to a 10% SDS-polyacrylamide gel, electrophoresed, and transferred to polyvinylidene difluoride membrane (PVDF) (Thermo Scientific, Rockford, IL, USA). Membranes were developed using Pierce ECL Western blotting substrate (Thermo Scientific). The following antibodies were used for immunoblotting: AKT, phosphorylated AKT (Ser473), mTOR, phosphorylated mTOR and β-actin, purchased from Cell Signaling Technology, MA, USA; and human ENT1 from Spring Bioscience, Pleasanton, CA, USA.

### Isolation of RNA and reverse transcription-polymerase chain reaction (RT-PCR)

Total RNA was extracted using Trizol (Invitrogen) as described by the manufacturer. Complementary DNA was subsequently synthesized from total cellular RNA using MMLV reverse transcriptase (Promega, Madison, WI, USA) and PCR was performed using a PCR thermal cycler (Labnet International Inc. Edison, NJ, USA). The PCR program used to amplify hENT1 and GAPDH consisted of a pre-cycle of 5 minutes at 94°C, 30 seconds at 30°C and 30 seconds at 72°C. Following this initial cycle, the reaction was continued for 26 cycles of 30 seconds at 94°C, 30 seconds at 60°C, and 30 seconds at 72°C and concluded with 5 minutes at 72°C. The primers sequences were as follows: hENT1 forward primer: 5′-ATCTGCGCTATTGCCAGTG-3′; hENT1 reverse primer: 5′-TCCAACTTGGTCTCCTGCTC-3′; GAPDH forward primer: 5′-CACCCAGAAGACTGTGGATGG-3′; GAPDH reverse primer: 5′-CCACCAC-CCTGTTGCTGTAG-3′.

### ENT1 activity

The ENT1 activity assay was performed as described previously [41], using a sodium free transport assay buffer (20 mM TrisHCl (pH 7.5), 3 mM K_2_HPO_4_, 1 mM MgCl_2_ 6 H_2_O, 2 mM CaCl_2_, 5 mM glucose and 130 mM N-methyl-D-glucamine (NMDG, pH 7.4). Briefly, cells were washed once with transport assay buffer and then suspended in transport assay buffer. After a pre-incubation with 1 μM NBMPR or vehicle (DMSO) for 15 minutes, uptake assays were started by adding equal volume of transport buffer containing 2 μM cold adenosine, [^3^H]-adenosine 4 μCi/ml plus NBMPR or DMSO. Time course of uptake under these conditions was performed to determine linearity (not shown). Uptake was stopped after 5 minutes followed by five rapid washes with ice cold transport buffer containing 1 mM unlabeled adenosine. The cell pellets were lysed in a lysis buffer containing 20 mM TrisHCl (pH7.5), 137 mM NaCl, 1% Triton X-100, 10% glycerol and 2 mM EDTA. After centrifugation at 1000 rpm for 10 minutes at 4°C, 80% of the SN was used to measure incorporated radioactivity and 20% to measure total protein content. Difference between total transport and transport in the presence of 1 μM NBMPR was defined as ENT1-adenosine transport.

### ENT1 cell surface detection

ENT1 present at the cell surface was assessed by a cell surface biotinylation assay with EZ-Link Sulfo-NHS-Biotin followed by precipitation with Neutroavidin-Agarose and immunoblot, as previously described [13].

### Statistical analysis

All data are given as means ± S.D. of at least three independent experiments. Comparison of treatments against controls was made using one-way Analysis of variance (ANOVA) followed by Bonferroni's least significant difference post hoc test. Data with two groups to compare were analyzed using a t-test. Survival curves were generated using the method of Kaplan and Meier and analyzed by the log-rank (Mantel-Cox) test. Statistical analysis was performed using Graph Pad Prism 5 statistical package. The significance level chosen for the statistical analysis was p < 0.05.
